# Erfassung und apparatives Monitoring des Ernährungsstatus von Patient*innen auf der Intensiv- und Intermediate Care Station: Positionspapier der Sektion Metabolismus und Ernährung der Deutschen Interdisziplinären Vereinigung für Intensiv- und Notfallmedizin (DIVI)

**DOI:** 10.1007/s00063-022-00918-4

**Published:** 2022-04-28

**Authors:** Arved Weimann, Wolfgang H. Hartl, Michael Adolph, Matthias Angstwurm, Frank M. Brunkhorst, Andreas Edel, Geraldine de Heer, Thomas W. Felbinger, Christiane Goeters, Aileen Hill, K. Georg Kreymann, Konstantin Mayer, Johann Ockenga, Sirak Petros, Andreas Rümelin, Stefan J. Schaller, Andrea Schneider, Christian Stoppe, Gunnar Elke

**Affiliations:** 1 Abteilung für Allgemein‑, Viszeral- und Onkologische Chirurgie, Klinikum St. Georg gGmbH, Delitzscher Str. 141, 04129 Leipzig, Deutschland; 2 Klinik für Allgemein‑, Viszeral- und Transplantationschirurgie, Ludwig-Maximilians-Universität München – Klinikum der Universität, Campus Großhadern, München, Deutschland; 3 Universitätsklinik für Anästhesiologie und Intensivmedizin und Stabsstelle Ernährungsmanagement, Universitätsklinikum Tübingen, Tübingen, Deutschland; 4 Medizinische Klinik und Poliklinik IV, Ludwig-Maximilians-Universität München – Klinikum der Universität, Campus Innenstadt, München, Deutschland; 5 Zentrum für Klinische Studien, Klinik für Anästhesiologie und Intensivtherapie, Universitätsklinikum Jena, Jena, Deutschland; 6 Klinik für Anästhesiologie mit Schwerpunkt operative Intensivmedizin, Charité Universitätsmedizin Berlin, Berlin, Deutschland; 7 Zentrum für Anästhesiologie und Intensivmedizin, Klinik für Intensivmedizin, Universitätsklinikum Hamburg-Eppendorf, Hamburg, Deutschland; 8 Klinik für Anästhesiologie, Operative Intensivmedizin und Schmerztherapie, Kliniken Harlaching und Neuperlach, Städtisches Klinikum München GmbH, München, Deutschland; 9 Klinik für Anästhesiologie, operative Intensivmedizin und Schmerztherapie, Universitätsklinikum Münster, Münster, Deutschland; 10 Kliniken für Anästhesiologie und Operative Intensivmedizin und Intermediate Care, Uniklinik RWTH Aachen, Aachen, Deutschland; 11 Flemingstraße 2, 22299 Hamburg, Deutschland; 12 Klinik für Pneumologie und Schlafmedizin, St. Vincentius-Kliniken, Karlsruhe, Deutschland; 13 Medizinische Klinik II, Klinikum Bremen Mitte, Bremen, Deutschland; 14 Interdisziplinäre Internistische Intensivmedizin, Universitätsklinikum Leipzig, Leipzig, Deutschland; 15 Anästhesie, Intensivmedizin und Notfallmedizin, Helios St. Elisabeth-Krankenhaus Bad Kissingen, Bad Kissingen, Deutschland; 16 Klinik für Gastroenterologie, Hepatologie und Endokrinologie, Medizinische Hochschule Hannover, Hannover, Deutschland; 17 Klinik und Poliklinik für Anästhesiologie, Intensivmedizin, Notfallmedizin und Schmerztherapie, Universitätsklinikum Würzburg, Würzburg, Deutschland; 18 Klinik für Anästhesiologie und Operative Intensivmedizin, Universitätsklinikum Schleswig-Holstein, Campus Kiel, Arnold-Heller-Straße 3, 24105 Kiel, Deutschland

**Keywords:** Malnutrition, Kritische Erkrankung, Ernährung, Intensivmedizin, Leitlinie, Malnutrition, Critical illness, Nutrition, Intensive care, Guideline

## Abstract

Die Erhebung des Ernährungsstatus zum Zeitpunkt der Aufnahme im Intensiv- oder Intermediate Care Bereich hat sowohl prognostische als auch therapeutische Relevanz im Hinblick auf die Planung einer individualisierten medizinischen Ernährungstherapie (engl. „medical nutrition therapy“, MNT). Diese Planung wird im Rahmen der Erstversorgung eines vital bedrohlichen Krankheitsbilds nachvollziehbar nicht priorisiert, jedoch im weiteren Verlauf häufig auch oft nicht mehr angemessen durchgeführt. Vor allem bei längerer Verweildauer besteht das Risiko einer Mangelernährung mit Aufbau eines kumulativen, prognoserelevanten Makro- und/oder Mikronährstoffdefizits. Bisher gibt es für Patient*innen auf Intensiv- und Intermediate Care Einheiten keine strukturierten Empfehlungen zur Erhebung des Ernährungsstatus. Das vorliegende Positionspapier der Sektion Metabolismus und Ernährung der Deutschen Interdisziplinären Vereinigung für Intensiv- und Notfallmedizin (DIVI) beinhaltet konsensbasierte Empfehlungen zur Erfassung und zum apparativen Monitoring des Ernährungsstatus von Patient*innen auf Intensiv- und Intermediate Care Stationen. Diese Empfehlungen ergänzen die aktuelle S2k-Leitlinie „Klinische Ernährung in der Intensivmedizin“ der Deutschen Gesellschaft für Ernährungsmedizin (DGEM) und der DIVI.

**Zusatzmaterial online:**

Im Zusatzmaterial dieses Beitrags (10.1007/s00063-022-00918-4) finden Sie die Angaben aller Autor*innen zum Interessenkonflikt.

## Abstract (en)

At the time of admission to an intensive or intermediate care unit, assessment of the patients’ nutritional status may have both prognostic and therapeutic relevance with regard to the planning of individualized medical nutrition therapy (MNT). MNT has definitely no priority in the initial treatment of a critically ill patient, but is often also neglected during the course of the disease. Especially with prolonged length of stay, there is an increasing risk of malnutrition with considerable prognostic macro- and/or micronutrient deficit. So far, there are no structured, evidence-based recommendations for assessing nutritional status in intensive or intermediate care patients. This position paper of the Section Metabolism and Nutrition of the German Interdisciplinary Association for Intensive and Emergency Medicine (DIVI) presents consensus-based recommendations for the assessment and technical monitoring of nutritional status of patients in intensive and intermediate care units. These recommendations supplement the current S2k guideline “Clinical Nutrition in Intensive Care Medicine” of the German Society for Nutritional Medicine (DGEM) and the DIVI.

## 1. Präambel

Für die Planung einer individualisierten medizinischen Ernährungstherapie (engl. „medical nutrition therapy“, MNT) auf der Intensiv (ITS)- oder Intermediate Care Station (IMC) ist die Erhebung des Ernährungsstatus eine wichtige Voraussetzung. Ernährungsmedizinische Diagnostik wird in der Erstversorgung eines vital bedrohlichen Krankheitsbilds nachvollziehbar nicht priorisiert, jedoch im weiteren Verlauf häufig auch nicht mehr nachgeholt. Dadurch besteht vor allem bei längerer Verweildauer das Risiko einer Mangelernährung mit Aufbau eines kumulativen, prognoserelevanten Energie‑, Protein- und Mikronährstoffdefizits [1]. Eine standardisierte Diagnostik der Mangelernährung kann grundsätzlich Patient*innen mit einem erhöhtem Risiko für einen längeren Aufenthalt auf der ITS bzw. IMC oder generell im Krankenhaus und mit einer erhöhten Sterblichkeit identifizieren [2]. Theoretisch können aus ernährungsmedizinischer Sicht 3 Patient*innengruppen unterschieden werden, wobei jedoch bis heute nicht sicher ist, wie diese Gruppen in der Praxis genau zu charakterisieren sind [3]:

- Patient*innen, bei denen durch eine MNT eine Multiorgandysfunktion und ein komplikationsreicher Verlauf nicht zu verhindern oder zu durchbrechen sein wird;
- Patient*innen, die sich rasch erholen, und keine MNT benötigen, da sie selbständig und frühzeitig wieder bedarfsdeckende orale Kost zu sich nehmen können;
- Patient*innen, deren klinischer Verlauf signifikant durch eine individualisierte MNT positiv beeinflusst werden kann.

Zur Identifikation dieser letztgenannten Subgruppe steht eine Reihe von Parametern zur Verfügung, deren Nutzen im Folgenden diskutiert werden soll.

## 2. Ziele des Positionspapiers

Im Sinne der Mission 2030 der Deutschen Interdisziplinären Vereinigung für Intensiv- und Notfallmedizin (DIVI) und in Ergänzung zur aktuellen S2k-Leitlinie „Klinische Ernährung in der Intensivmedizin“ der Deutschen Gesellschaft für Ernährungsmedizin (DGEM) aus dem Jahr 2018 [4] sollen mit 2 Positionspapieren der DIVI-Sektion „Metabolismus und Ernährung“ die Kompetenz und Qualität weiterentwickelt werden. Während dieses erste Positionspapier verschiedene Möglichkeiten der Erhebung und des (apparativ) technischen Monitorings des Ernährungsstatus vorstellt und diskutiert, wird das zweite Positionspapier laborchemische Parameter zur Beurteilung des Ernährungsstatus und der metabolischen Toleranz sowie die Messung des Energieumsatzes umfassen.

„Intensivstation“ und „Intermediate Care Station“ werden entsprechend der DIVI-Strukturempfehlungen definiert [5, 6]. Die Empfehlungen sollen das Bewusstsein für die prognostische und mögliche therapeutische Relevanz des Ernährungsstatus für die Planung einer individualisierten MNT fördern und die strukturellen Anforderungen der DIVI an die apparative Ausstattung einschließlich der bildgebenden Diagnostik ergänzen [5, 6].

Da der Operationen- und Prozedurenschlüssel(OPS)-Code 8-98j „Ernährungsmedizinische Komplexbehandlung“ auch bei Patient*innen auf der ITS oder IMC Anwendung finden kann, werden im Folgenden die Vorgaben zur Kodierung berücksichtigt [7].

## 3. Methodisches Vorgehen und Konsensfindung

Im Rahmen eines Onlinesymposiums der DGEM in Zusammenarbeit mit der DIVI-Sektion Metabolismus und Ernährung am 20./21.11.2020 wurde der Themenkomplex definiert und anhand der Präsentationsinhalte gemeinsam ein erster Textentwurf erstellt. Zwischenzeitlich erfolgte eine aktuelle Literatursuche anhand der auch für die Leitlinie verwendeten Schlüsselwörter und Suchstränge. Diese wurde im weiteren Verlauf mehrfach aktualisiert. Der Textentwurf wurde den Sektionsmitgliedern am 31.01.2021 zur Durchsicht vorgelegt und nachfolgend in einer Onlinesitzung am 15.02.2021 strukturiert diskutiert. Es folgten analog weitere Überarbeitungen mit Onlinesitzung am 18.05.2021, danach die Erstellung des Texts, der den Sektionsmitgliedern am 13.12.2021 vorgelegt wurde. Die abschließende Diskussion und Konsensusfindung erfolgte am 21.12.2021 online. Für die Empfehlungen bestand jeweils 100 %ige Zustimmung. Der überarbeitete Text wurde am 23.12. und 30.12.2021 zur Durchsicht an die Sektionsmitglieder und anschließend an das DIVI-Präsidium versandt. Am 08.02.2022 wurde das vorliegende Positionspapier im Rahmen der DIVI-Präsidiumssitzung (online) in seiner finalen Version verabschiedet.

## 4. Erhebung des Ernährungszustands

Grundsätzlich erfolgt bei Aufnahme auf eine ITS oder IMC eine allgemeine Risikoeinschätzung anhand etablierter Scores wie z. B. dem Acute Physiology and Chronic Health Evaluation (APACHE II) Score oder dem Sepsis-related Organ Failure Assessment (SOFA) Score. Diese klassischen Scores berücksichtigen jedoch nicht den Ernährungszustand der Patient*innen. Da bei bis zu 80 % der kritisch kranken Patient*innen bereits bei Aufnahme eine Mangelernährung vorliegt oder sich innerhalb des Verlaufs auf der ITS einstellen kann [8], erscheint es grundsätzlich sinnvoll, den Ernährungsstatus in die Risikoeinschätzung für einen verlängerten Aufenthalt, die Entwicklung einer (Multi‑)Organdysfunktion und eine prolongierte Rehabilitation miteinzubeziehen.

Dadurch sollte sich die prognostische Sicherheit erhöhen und es sollte zumindest möglich sein, diejenigen Patient*innen zu identifizieren, die von einer besonders sorgfältigen Überwachung und leitliniengerechten Ernährung profitieren [4]. Eine simple Steuerung der Kalorien‑/Proteinzufuhr (vor allem in der Akutphase der kritischen Erkrankung) anhand des Ernährungszustands (nach dem Motto „je schlechter, umso mehr“) ist inzwischen jedoch verlassen worden. Eine inadäquat überwachte, „aggressive“ Kalorienzufuhr in der Akutphase kann unter Umständen die Morbidität sogar erhöhen und die Verweildauer verlängern [9, 10]. Besonders in dieser Phase ist eine nach der metabolischen und gastrointestinalen Toleranz ausgerichtete individuelle MNT und Steigerung der Substratzufuhr vorzunehmen [4].

### 4.1. Mangelernährung als Risiko

In einer überernährten Gesellschaft werden für ITS- und IMC-Patient*innen bzw. generell für hospitalisierte Patient*innen die Risiken einer Mangelernährung unterschätzt. Mogensen et al. [11] konnten anhand einer retrospektiven Analyse in einem Kollektiv von 6518 kritisch kranken Patient*innen zeigen, dass eine Protein-Energie-Mangelernährung bei Aufnahme – definiert als krankheitsbedingte Gewichtsabnahme, Untergewicht, Verlust an Muskelmasse und verminderte Energie- oder Proteinaufnahme – im Vergleich zu nicht-mangelernährten Patient*innen mit einem doppelt so hohen Sterblichkeitsrisiko assoziiert war. Grundsätzlich ist eine vorbestehende Mangelernährung in Verbindung mit einem akuten Organversagen ein unabhängiger, signifikanter Prädiktor für eine schlechtere Prognose [8]. Diese Assoziation hat sich relevant auch bei intensivpflichtigen COVID-19-Patient*innen gezeigt [12, 13]. Fast zwei Drittel dieser Patient*innen besaßen bei Aufnahme ein deutlich erhöhtes ernährungsmedizinisches Risiko (Nutritional Risk Screening Score [NRS] ≥ 5), das mit einer Letalität von 87 % assoziiert war. Patient*innen mit niedrigeren NRS-Score-Werten starben signifikant seltener (49 %, *p* < 0,01) [13].

### 4.2. Adipositas-Paradox

Häufig wird auf der Basis des sog. Adipositas-Paradox auf einen Überlebensvorteil für (speziell auch septische) Intensivpatient*innen hingewiesen, die übergewichtig oder sogar adipös sind [14, 15]. In einer aktuellen Metaanalyse (8 Studien bei 9696 septischen Patient*innen) fand sich im Vergleich zu Normalgewichtigen ein signifikant besseres Überleben nur bei Übergewichtigen mit einem Body Mass Index (BMI) von 25–30 kg/m^2^, nicht jedoch bei Adipositas (BMI 30–40 kg/m^2^) oder morbider Adipositas (BMI > 40 kg/m^2^) [16]. Eine „Dose-Response“-Metaanalyse von 31 Studien mit 238.961 Patient*innen konnte nur bis zu einem BMI ≤ 35 kg/m^2^ protektive Wirkungen identifizieren, während ein BMI > 35 kg/m^2^ mit einer signifikant erhöhten Letalität einherging [17]. In einer Studienkohorte von 6357 Patient*innen konnten keine signifikanten Vorteile für kritisch kranke Adipositaspatient*innen (18,9 % der Fälle) identifiziert werden [18].

Somit scheinen adipöse Patient*innen nicht speziell vor den negativen Folgen einer Sepsis geschützt zu sein. Mögliche Vorteile bei Übergewichtigen beruhen aus metabolischer Sicht nicht auf der erhöhten Fettmasse per se, sondern ergeben sich indirekt durch eine sekundäre Erhöhung der fettfreien Masse bzw. Muskelmasse, die mit der erhöhten Fettmasse einhergehen kann [19].

### 4.3. Definition der Mangelernährung

Die 2019 von den internationalen Fachgesellschaften konsentierte Global-Leadership-Initiative-on-Malnutrition(GLIM)-Definition der Mangelernährung beinhaltet phänotypische und ätiologische Kriterien, die auch für Patient*innen im ITS- oder IMC-Bereich angewandt werden können [20, 21]. Phänotypische Kriterien sind: unfreiwilliger Gewichtsverlust, niedriger BMI, verminderte Muskelmasse und anamnestisch verminderte Nahrungsaufnahme oder -resorption sowie Schwere der Grunderkrankung/Inflammation. Jeweils ein ätiologisches oder phänotypisches Kriterium muss erfüllt sein, damit eine Mangelernährung klinisch diagnostiziert werden kann [16]. Biochemisch entspricht dies einem abnormen Verhältnis von Gesamtkörpereiweißmasse (überwiegend Muskelmasse) zu Gesamtkörpergewicht und einem Defizit an Mikronährstoffen.

Als Ursachen für eine Malnutrition werden unterschieden [22]:

- bedingt durch eine akute Erkrankung: akute Inflammation mit schwerem Ausmaß (Sepsis, Verbrennung, Trauma).
- ernährungsbedingt, z. B. chronischer Hungerzustand ohne Inflammation, Nahrungskarenz (z. B. sozioökonomisch, prolongierte perinterventionelle Nüchternheit).
- bedingt durch eine chronische Erkrankung: chronische Inflammation mit mildem oder moderatem Ausmaß (z. B. Malabsorption bei chronischer Pankreatitis, chronisch-entzündlicher Darmerkrankung, Pankreaskarzinom, rheumatoide Arthritis, sarkopene Adipositas).

Alle 3 Formen können sich bei Patient*innen im ITS- oder IMC-Bereich überlagern.

### 4.4. Medizinische Ernährungstherapie (MNT) und Ernährungszustand

Die DGEM-Leitlinie empfiehlt mit starkem Konsens: „Der Ernährungszustand sollte zum Zeitpunkt der Aufnahme auf die Intensivstation abgeschätzt werden“ [4]. Hierbei ist die Einschätzung des Ernährungszustands zuallererst Ausdruck einer besonderen Sorgfalt im Rahmen der MNT. Auch für elektive präoperative/präinterventionelle Patient*innen ohne Organdysfunktion, jedoch mit einer hohen Wahrscheinlichkeit für eine postoperative/postinterventionelle intensivmedizinische oder IMC-Therapie wird empfohlen, den Ernährungsstatus – wenn nicht bereits auf der Basis der allgemeinen Patient*innenaufnahme vorliegend – spätestens im Rahmen des Prämedikationsgesprächs zu erheben.

Mangelernährte Patient*innen benötigen eine gründliche Beachtung der Indikation bzw. der individuellen metabolischen Toleranz einer MNT. So bedarf eine MNT insbesondere auch eines adäquaten Monitorings, das zusätzlich eine Verlaufskontrolle des Ernährungsstatus mit einbeziehen sollte [23, 24].

Die MNT muss immer individuell im Kontext von akuter Inflammation, metabolischem Zustand, Organfunktion und Körperzusammensetzung bei Aufnahme und im weiteren Krankheitsverlauf gesehen werden und risiko- bzw. phasenadaptiert gesteuert durch die individuelle metabolische Toleranz erfolgen [4].

Ziel der MNT muss es grundsätzlich sein, einerseits ein Kalorien‑, Protein- und auch ein Mikronährstoffdefizit zu vermeiden bzw. eine vorbestehende Malnutrition nicht zu verstärken; andererseits sollte jedoch die Prognose nicht durch eine unangepasste, zu aggressive Substratzufuhr verschlechtert werden. Diese Ziele sind besonders relevant für

- bereits primär bei Aufnahme mangelernährte Risikopatient*innen;
- Patient*innen, die aufgrund ihrer Grunderkrankung ein hohes Risiko aufweisen, während ihres Aufenthaltes auf der ITS oder IMC bzw. generell während ihrer Hospitalisation eine Mangelernährung als zusätzliches Risiko zu entwickeln.

#### Empfehlung 1

Sofern nicht bereits vorliegend soll die Einschätzung des Ernährungszustands zum Zeitpunkt der Aufnahme auf der Intensiv- oder Intermediate Care Station erfolgen.

## 5. Methoden zur Bestimmung des Ernährungszustands

### 5.1 Anthropometrie

Die präzise klinische Erfassung des Ernährungsstatus anhand klassischer Parameter (speziell Gewicht und BMI) kann insbesondere bei Intensivpatient*innen zum Zeitpunkt der Aufnahme (und auch im Verlauf) durch beträchtliche interstitielle Flüssigkeitseinlagerungen („capillary leak“) erschwert sein. Die je nach Stadium der Erkrankung zu beobachtende Hyperhydratation macht das aktuelle Körpergewicht und den mithilfe der Körpergröße errechneten BMI zu unzuverlässigen Parametern bei der Beurteilung des Ernährungsstatus. So korreliert die Messung des Körpergewichts unabhängig von der Phase der Erkrankung nicht nur mit der Eiweißmasse, sondern auch mit dem Hydratationszustand der Patient*innen.

Das „gewohnte“ Körpergewicht und ein eventueller Gewichtsverlust vor Aufnahme können insbesondere bei Notfallpatient*innen nur fremdanamnestisch und mit dem Risiko der Fehleinschätzung in Erfahrung gebracht werden. Die Messung des aktuellen Körpergewichts kann aufwändig sein. Eine Bettwaage steht nicht immer zur Verfügung.

Trotz dieser methodischen Limitationen sollte dennoch bei allen Patient*innen im ITS- und IMC-Bereich die regelmäßige Messung des aktuellen Körpergewichts möglich sein und bei mobilisierbaren Patient*innen durch eine konventionelle Personen- bzw. Stuhlwaage (alternativ Bettwaage) erfolgen. Die möglichst genaue Erfassung des Körpergewichts unter Berücksichtigung der Flüssigkeitsbilanz und des Hydratationsstatus ist dabei nicht nur Grundlage für die Kalkulation des BMI, sondern auch für medikamentöse Therapien [25].

Ein einfacher anthropometrischer Parameter ist der mittlere Armumfang, der sich bei Patient*innen mit einem Köpergewicht < 15. Perzentile als ein Prädiktor für das Risiko schwerer Komplikationen und erhöhter Letalität erwiesen hat [26].

Bei der Messung des oberen Bauchumfangs zur Abschätzung der viszeralen Adipositas mit CT bei COVID-19-Patient*innen war jeder Zentimeter Zunahme mit einer 1,13-fach höheren Wahrscheinlichkeit für eine Intensivbehandlung und einer 1,25-fach höheren Wahrscheinlichkeit für eine mechanische Beatmung assoziiert [27].

Bei kooperationsfähigen Patient*innen ohne vorbestehende oder erworbene neurologische Komorbidität (Critical-illness-Polyneuropathie, oder -Myopathie) stellt die Dynamometrie mit Messung der Handkraft eine einfache und quantifizierbare Methode zur funktionellen (Verlaufs‑)Kontrolle der Muskelkraft dar [28]. Die Handgriffstärke ist signifikant niedriger bei Langzeitintensivpatient*innnen als bei Patient*innen mit kurzer Behandlungsdauer und korreliert nach mechanischer Beatmung mit der Rate an Reintubationen [29–31]. Eine Assoziation mit der sonographisch bestimmten Querschnittsfläche des M. rectus femoris ist bei Patient*innen mit Sepsis gezeigt worden [32]. Voraussetzung für die Messung ist allerdings eine standardisierte Untersuchungstechnik ohne patientenseitige Limitationen. Da klare Cut-off-Werte für Intensivpatient*innen fehlen, liegt der Wert vor allem in der intraindividuellen Verlaufskontrolle. Für die ernährungsmedizinische Komplexbehandlung OPS 8-98j ist die Dynamometrie als Verlaufskontrolle anerkannt.

#### Empfehlung 2

Unter Berücksichtigung der methodischen Limitationen sollte bei allen Intensiv- und Intermediate Care-Patient*innen die regelmäßige Messung des aktuellen Körpergewichts möglich sein und mittels Bettwaage bzw. bei mobilisierbaren Patient*innen durch eine konventionelle Personen- bzw. Stuhlwaage erfolgen.

Zur Prüfung der Muskelkraft und funktionellen Verlaufskontrolle wird bei kooperationsfähigen Patient*innen die serielle Dynamometrie mit Messung der Handkraft empfohlen.

### 5.2 Scores zur Erhebung des Ernährungszustands

Die verfügbaren und ansonsten bewährten Screening- und Messinstrumente sind für die Erhebung des Ernährungsstatus im ITS- und IMC-Bereich nicht gut validiert. Die Befunde weisen eine große Unsicherheit auf. Bei fehlender Evidenz geben deswegen die international verfügbaren Leitlinien keine klaren Empfehlungen im Hinblick auf die präzise Methodik bzw. den Umfang personeller Ressourcen, die zur Erhebung des Ernährungszustands vorgehalten werden sollen [33–36]. Dieses Defizit ist auch in einer aktuellen Leitlinienübersicht noch einmal deutlich geworden [37].

Die Tab. 1 zeigt eine Übersicht der aktuellen Leitlinienempfehlungen, Tab. 2 eine Übersicht der Screeningtools zur Einschätzung des Ernährungszustands.

**Table 1:** 

Fachgesellschaft	Parameter
DGEM Leitlinie „Intensivmedizin“ [4]	Einschätzung des Ernährungszustands zum Zeitpunkt der Aufnahme mit DGEM-Kriterien oder Subjective Global Assessment (SGA)
	Nichtinvasive serielle Untersuchungen der Skelettmuskelmasse mittels Sonographie/MRT/CT zum Aufnahmezeitpunkt als auch während des Aufenthalts auf der Intensivstation
DGEM Leitlinie „Terminologie in der Klinischen Ernährung“ [38]	BMI < 18,5 kg/m^2^ oder
	Ungewollter Gewichtsverlust > 10 % in den letzten 3–6 Monaten oder
	BMI < 20 kg/m^2^ und ungewollter Gewichtsverlust > 5 % in den letzten 3–6 Monaten oder
	Nahrungskarenz > 7 Tage
A.S.P.E.N. Leitlinie „Intensivmedizin“ [26]	Risikoabschätzung mit validiertem Score, Nutritional Risk Score (NRS), Nutrition Risk in the Critically Ill (NUTRIC)
	Unzureichende Energieaufnahme
	Gewichtsverlust
	Verlust von Muskelmasse und subkutanem Fettgewebe
	Wassereinlagerungen
	Verminderter funktioneller Status
ESPEN Leitlinie „Intensivmedizin“ [28]	Anamnese und klinische Untersuchung
	Verminderter BMI
	Unfreiwilliger Gewichtsverlust
	Körperzusammensetzung mit Muskelmasse und -kraft – wenn möglich

*A.S.P.E.N.* American Society for Parenteral and Enteral Nutrition, *BMI* Body Mass Index, *CT* Computertomographie, *DGEM* Deutsche Gesellschaft für Ernährungsmedizin, *ESPEN* European Society for Clinical Nutrition and Metabolism, *MRT* Magnetresonanztomographie

**Table 2:** 

Screening-Tool	Parameter
NUTRIC (Nutrition Risk in Critically Ill)	Alter
	APACHE (Acute Physiology and Chronic Health Evaluation) II-Score
	SOFA (Sepsis-related Organ Failure Assessment)-Score
	Anzahl Komorbiditäten
	Verweildauer im Krankenhaus vor ITS-Aufnahme
	IL‑6 (Interleukin-6) (fakultativ)
NRS-2002 (Nutritional Risk Screening)	BMI ≤ 20,5 kg/m^2^
	Gewichtsverlust > 5 % während der letzten 3 Monate
	Verminderte Nahrungsaufnahme
	Schweregrad der Erkrankung
MUST (Malnutrition Universal Screening Tool)	BMI
	Gewichtsverlust
	Schweregrad der Erkrankung
SGA (Subjective Global Assessment)	Anamnese – Gewicht – Nahrungsaufnahme – Gastrointestinale Symptome – Funktioneller Status
	Körperliche Untersuchung – Subkutanes Fettgewebe – Muskelmasse – Ödeme – Aszites
MNA (Mini Nutritional Assessment)	BMI oder Wadenumfang (in cm)
	Anamnese – Abnahme der Nahrungsaufnahme in den letzten 3 Monaten durch Appetitverlust, Verdauungsprobleme, Kau‑/Schluckstörungen – Gewichtsverlust in den letzten 3 Monaten – Mobilität – Akute Krankheit oder psychischer Stress – Neuropsychologische Probleme wie Demenz, Depression
	Voraussichtliche Nahrungskarenz von mehr als 5 Tagen und akute Erkrankung

*BMI* Body Mass Index, *ITS* Intensivstation

### 5.3. NRS, MNA, SGA

Die DGEM gibt auf ihrer Webseite einen Überblick zu den empfohlenen Screeningmethoden inklusive der jeweiligen Screeningfragebögen (www.dgem.de/screening), die zur Abklärung einer Mangelernährung zur Verfügung stehen.

Die European Society for Clinical Nutrition and Metabolism (ESPEN) empfiehlt für nicht-intensivpflichtige Patient*innen ein 2‑stufiges Konzept unter Verwendung des gut validierten Nutritional Risk Score (NRS). Wird eine der in Tab. 2 aufgeführten Fragen des Vorscreenings mit „ja“ beantwortet, schließt sich das Hauptscreening an, in dem die Störung des Ernährungszustands und die Krankheitsschwere anhand eines graduellen Punktescores genauer quantifiziert werden (zusätzlich 1 Punkt, wenn Alter ≥ 70 Jahre). Scorewerte > 3 charakterisieren eine Risikopatient*in, Werte ≥ 5 eine Hochrisikopatient*in mit den Zeichen der manifesten Mangelernährung [39].

Das Mini Nutritional Assessment (MNA) ist ein Screeninginstrument zur Erfassung des Ernährungszustands bei Individuen > 65 Jahre in der häuslichen Pflege, Krankenhaus oder Pflegeheim und besitzt ebenfalls einen 2‑stufigen Aufbau: Voranamnese mit 6 Items (A–F) mit max. 14 Punkten, und Anamnese mit weiteren 12 Items (G–R) – davon 2 anthropometrische Messungen (Oberarmumfang und Wadenumfang) – mit max. 16 Punkten [40].

Das Subjective Global Assessment (SGA) ist eine einfache, reproduzierbare bettseitige Methode, die auf der Grundlage von anamnestischen (Gewichtsveränderung, Nahrungszufuhr, gastrointestinale Symptome, Leistungsfähigkeit, Grunderkrankung) und klinischen Untersuchungsbefunden (Unterhautfettgewebe, Muskelmasse, Ödeme) den Ernährungszustand der Patient*in quantifiziert [41]. Im Hinblick auf die Früherkennung einer Mangelernährung scheint das aufwändigere SGA den Screeningverfahren überlegen zu sein [42]. Daten liegen auch für Intensivpatient*innen vor, bei denen eine mit dem SGA diagnostizierte Mangelernährung mit einer höheren Wiederaufnahmerate auf die ITS, einer längeren Krankenhausverweildauer und auch höheren Krankenhausletalität einherging [43, 44].

Der NRS wurde in einer Studie an 260 älteren internistischen und chirurgischen Intensivpatient*innen > 65 Jahre evaluiert. Es fand sich eine NRS-definierte Häufigkeit der Mangelernährung von 23–34 % [45]. Im Vergleich mit dem MNA zeigte der NRS die höchste Sensitivität, während SGA und MNA Short Form eine höhere Spezifität aufwiesen. Eine mit diesen Methoden identifizierte Mangelernährung war signifikant mit einem längeren Krankenhausaufenthalt, einem höheren Bedarf an poststationärer pflegerischer Unterstützung und auch einer höheren Letalität assoziiert.

In einer Metaanalyse von 20 Studien mit 1168 Patient*innen fand sich unter Verwendung von NRS, SGA und MNA eine Prävalenz der Mangelernährung von 38–78 % [7]. Das SGA war prädiktiv dem MNA klar überlegen. Die Assoziation zwischen Screening und Risiko für Mangelernährung war hingegen wenig konsistent. Es bestand eine unabhängige Assoziation zwischen dem Vorliegen einer Mangelernährung und einer schlechteren Prognose. Weiteres Ergebnis dieser Analyse war, dass zur Abschätzung der Ernährungsstatus-assoziierten Prognose SGA und MNA besser geeignet waren als der NRS [7]. Im Vergleich zum Grad C des SGA als Goldstandard für die Diagnose einer Mangelernährung hat der NRS in einer anderen Studie bei Intensivpatient*innen eine höhere Sensitivität (79,1 %) und Spezifität (94,8 %) gezeigt [46].

Aus Sicht der Arbeitsgruppe sind diese Scores jedoch nur begrenzt für den Einsatz bei kritisch kranken Patient*innen geeignet, da sie eine Anamnese voraussetzen, die zuverlässig nur bei wachen und auskunftsfähigen Patient*innen zu erheben ist. Fremdanamnestische Angaben sind in der klinischen Routine oft nur aufwändig zu erheben bzw. unzuverlässig.

Zusätzlich limitierend ist, dass der NRS in seinem Scoresystem eine kritische Erkrankung immer mit 3 Punkten bewertet, wodurch alle kritisch kranken Patient*innen auf der ITS *per se* bei Aufnahme ein Risiko für eine Mangelernährung aufweisen und keine weitere Differenzierung möglich ist. Der SGA erfordert neben der Anamnese geschulte Untersucher*innen [41]. Der Nutzen des MNA wird dadurch eingeschränkt, dass er ursprünglich zur Erfassung des Ernährungszustands älterer, nicht kritisch kranker Patient*innen > 65 Jahre entwickelt wurde [47].

### 5.4. NUTRIC-Score

Der Nutrion Risk In Critically Ill (NUTRIC)-Score wurde speziell für Intensivpatient*innen entwickelt, um die Wahrscheinlichkeit für einen komplizierten Intensivverlauf („Langlieger“) und das damit assoziierte ernährungsmedizinische Risiko abschätzen zu können [48]. Der Score beruht u. a. auf dem APACHE II- und SOFA-Score. Ausgangspunkt ist ein primär nicht mangelernährter Patient, sodass keine typischen Parameter des Ernährungsstatus einfließen. Weitere Parameter neben dem APACHE II- und SOFA-Score sind Alter, Anzahl von Komorbiditäten sowie Krankenhausverweildauer vor Aufnahme auf die ITS. Die Variable Interleukin(IL)-6-Konzentration ist je nach Verfügbarkeit optional. Ein Score < 6 Punkte (bei Nichtverfügbarkeit der IL-6-Konzentration < 5) zeigt an, dass das Risiko für die Entwicklung einer Mangelernährung gering ist, ab einem Punktwert von 6 (bei Nichtverfügbarkeit der IL-6-Konzentration ab Punktewert 5) muss von einem erhöhten Risiko für einen komplizierten Verlauf mit längerem Aufenthalt auf einer Intensivstation ausgegangen werden [48].

In einer prospektiven Beobachtungsstudie an 2863 mechanisch beatmeten Intensivpatient*innen erfolgte eine Risikostratifizierung gemäß dem NUTRIC-Score [49]. Patient*innen mit hohem Risiko (NUTRIC-Score ≥ 5) und längerer Verweildauer > 12 Tagen profitierten von einer aggressiveren Energie- und Eiweißzufuhr (signifikant niedrigere 60-Tage-Letalität), während dies nicht für Patient*innen mit niedrigem Score und einer Verweildauer von ≤ 4 Tagen galt. Die Relevanz dieser Beobachtungen ist jedoch aufgrund methodischer Limitationen bei der Datenauswertung (keine Berücksichtigung des „confounding by indication“, von kompetitiven Risiken, der Zeitabhängigkeit der Nahrungszufuhr und von zeitvariierenden bzw. nichtlinearen Assoziationen) eingeschränkt.

In der explorativen Post-hoc-Analyse der randomisiert-kontrollierten PERMIT-Studie zum Vergleich einer permissiven Unterernährung und einer kalorienzielorientierten Ernährungstherapie wurde ein hohes ernährungsmedizinisches Risiko ab einem NUTRIC-Score > 4 angenommen, wobei die klinische Prognose unabhängig vom NUTRIC-Score war [50]. In einer weiteren Kohortenstudie (*n* = 312) ließ sich keine Assoziation zwischen dem NUTRIC-Score und dem NRS 2002 zeigen [51]. Lew et al. [52] verglichen einen modifizierten NUTRIC-Score mit dem SGA und fanden keine gute Übereinstimmung. Die Kombination beider Scores zeigte jedoch die beste Trennschärfe im Hinblick auf die Prognose (Letalität). In einer weiteren Post-hoc-Analyse der multizentrischen REDOXS-Studie zeigte sich dagegen eine signifikante Assoziation zwischen NUTRIC-Score und 28-Tage- und 6‑Monate-Letalität bei insgesamt 1199 kritisch kranken Patient*innen mit Multiorgandysfunktion (mittlerer NUTRIC-Score 5,5) [53].

Die 2016 publizierte Leitlinie der American Society for Parenteral and Enteral Nutrition (A.S.P.E.N.) empfiehlt auf Basis einer Expertenmeinung die Erhebung des NUTRIC-Scores bei Aufnahme auf die Intensivstation [34]. Die ESPEN-Leitlinie [36] empfiehlt eine Beurteilung des Ernährungszustands, ohne sich dabei auf ein bestimmtes Verfahren festzulegen; aus Sicht der DGEM/DIVI-Arbeitsgruppe ist der NUTRIC-Score nicht zur Abschätzung des Ernährungszustands geeignet, sondern reflektiert nur das durch Inflammation/Infektion und Multiorgandysfunktion beeinflusste Gesamtrisiko. Gerade dies erfasst hingegen der NRS überhaupt nicht [39]. Es gibt Hinweise dafür, dass die Erweiterung des NUTRIC-Scores um das Merkmal Sarkopenie (anhand der Messung des Wadenumfangs, „SARC-CALF“) und um das Merkmal Gebrechlichkeit vor Aufnahme („clinical frailty scale“) einen noch besseren Prädiktor für ein ungünstiges Outcome darstellen könnte („modified NUTRIC score SF“) [54]. Eine ausreichende Validierung ist jedoch noch ausstehend.

Da der NUTRIC-Score das Risiko kritisch Kranker insgesamt abbildet, empfiehlt sich die Kombination mit einer zusätzlichen ernährungsmedizinischen Untersuchung; z. B. Bestimmung der Körperzusammensetzung.

#### Empfehlung 3

Ein regelhaftes Screening mit NRS oder MNA wird nicht empfohlen. Die Durchführung des SGA ist zur Erfassung des Ernährungszustands für Patient*innen auf Intensiv- und Intermediate Care Stationen geeignet, setzt aber erfahrene Untersucher*innen voraus. Der NUTRIC-Score kann zur Identifikation von Patient*innen mit Risiko für eine längere Liegedauer eingesetzt werden.

### 5.5. Nicht-invasive Bestimmung der Skelettmuskelmasse als Surrogatvariable zur Erfassung des Ernährungszustands

In einer systematischen Übersicht von 6 Studien zum Muskelverlust von Intensivpatient*innen wurde in den ersten 14 Tagen nach Aufnahme ein Muskelmasseverlust von bis zu ca. 20 % beobachtet, wobei die Muskelmasse mittels γ‑Neutronen-Aktivierung, Computertomographie (CT) oder Ultraschall quantifiziert wurde [55]. Problematisch ist bei den in der klinischen Routine einsetzbaren computertomographischen oder sonographischen Verfahren bisher jedoch, dass speziell im Verlauf gleichbleibende Befunde nur scheinbar eine unveränderte Muskelmasse anzeigen können; Volumenverluste durch eine Abnahme der myofibrillären Masse können durch eine Volumenzunahme aufgrund einer interstitiellen Flüssigkeitseinlagerung („capillary leak“)/Bindegewebsvermehrung überdeckt werden.

Eine endgültige Bewertung des Nutzens bzw. der Zuverlässigkeit sonographischer Methoden oder der Bioimpedanzanalyse zur Erfassung der Körpermuskelmasse ist derzeit noch ausstehend [56, 57]. Trotzdem soll im Folgenden auch ein kurzer Überblick über die Charakteristika beider diagnostischer Verfahren gegeben werden.

#### 5.5.1. Muskelsonographie

Die Muskelsonographie ist ein nicht-invasives, bettseitig verfügbares Verfahren, mit dem ein Muskelverlust als Zeichen der Mangelernährung diagnostiziert werden kann und eine Schätzung der fettfreien Masse möglich ist. Zusätzlich ermöglicht das Verfahren die repetitive Messung und somit eine quantitative Verlaufsbeurteilung der Muskelmasse. Die Methode ist prinzipiell ohne besondere Vorkenntnisse in der Sonographie rasch erlernbar.

Eine einheitliche standardisierte Messvorgabe gibt es jedoch nicht, wobei allerdings typischerweise der M. quadrizeps femoris oder der M. rectus femoris im Querschnitt vermessen werden. Der Messpunkt für den M. rectus femoris liegt typischerweise auf der Linie von der Spina iliaca anterior superior zum proximalen Rand der Patella (mit einem Abstand von 60 % der Gesamtlänge zur Spina iliaca anterior superior). Vermessen werden die Querschnittsfläche des M. rectus femoris oder technisch einfacher die Quadrizepsdicke (Durchmesser) bestehend aus M. rectus femoris und M. vastus intermedius. Die Muskelsonographie weist bezogen auf Untersucher*in sowohl intra- als auch interindividuell eine hervorragende Reliabilität im Hinblick auf die Bestimmung der Muskeldicke und Querschnittsfläche auf [58–63].

So fanden Pardo et al. [64] bei 280 Messungen der Quadripzepsmuskeldicke an 29 Patient*innen eine gute intra- als auch interindividuelle Reliabilität, wenn die Messungen entweder in der Mitte (Korrelationskoeffizient 0,74 und 0,86) oder an der Zweidrittelstelle (0,83 und 0,81) der Messlinie erfolgten. Deswegen empfehlen die Autoren die Sonographie als Instrument zur Bestimmung des Ausgangszustands bei Aufnahme und zur Verlaufskontrolle der Effizienz einer MNT.

In einer multizentrischen Studie zur Validierung der Muskeldickenmessung an 149 Intensivpatient*innen ergab sich beim Vergleich von Sonographie und CT eine signifikante Korrelation von 0,45 [59]. In der Regressionsanalyse zur Güte der Vorhersage der Muskelmasse konnte eine signifikante Erhöhung des Konkordanzindex von 0,67 auf 0,77, jedoch nur durch die Einbeziehung zusätzlicher Variablen (Alter, Geschlecht, BMI, Charlson-Komorbiditätsindex und Zuordnung der Aufnahme [chirurgisch/internistisch]) erreicht werden. Aufgrund dieser Ergebnisse ist die Einmalmessung der Quadrizepsdicke ohne ergänzende Informationen wohl eher kritisch zu sehen.

Die Datenlage für die Querschnittsflächenmessung ist etwas besser; so korreliert der Quadrizepsquerschnitt sehr gut (Männer r = 0,88, Frauen r = 0,89) mit der Gesamtmuskelmasse im MRT [65]. Drei Studien konnten eine Assoziation zwischen Querschnittsfläche und den Ergebnissen funktioneller Messungen nachweisen, während dies nur in einer Studie für die Muskeldickenmessung gelang [60, 66, 67]. Eine weitere prospektiv-randomisierte Studie bei Langzeitintensivpatient*innen in der chronischen Phase konnte mittels sonographischer Verlaufskontrollen der Quadrizepsmuskeldicke den aus CT-morphologischen bzw. neutronenaktivierenden Untersuchungen bekannten Verlust an Muskelmasse bestätigen [68]. In 2 Beobachtungsstudien hat sich eine Assoziation zwischen dem sequenziellen Verlust der sonographisch gemessenen Quadrizepsdicke und der Krankenhaus- sowie 60-Tage-Letalität gezeigt [69, 70].

Limitierend für alle sonographischen Verfahren ist, dass Veränderungen des Hydratationszustands die Messergebnisse signifikant beeinflussen können [71, 72] und dass nicht die Quadrizepsmuskelmasse, sondern die lumbale Muskelmasse am besten mit der Gesamtkörpereiweißmasse korreliert [73].

#### 5.5.2. Computertomographie

Computertomographien des Abdomens werden bei Patient*innen im ITS- und IMC-Bereich mit kompliziertem Verlauf aus klinischen Gründen oftmals sogar mehrfach durchgeführt. Die CT-morphologischen Befunde können auch für eine Analyse der Körperzusammensetzung herangezogen werden. Die quantitative Analyse der Muskulatur im computertomographischen Querschnitt des Abdomens (CSA) im Bereich der Lendenwirbelsäule (meist auf Höhe von L3) korreliert gut mit der Muskel- und Fettmasse des Gesamtkörpers. Zur mathematischen Auswertung stehen verschiedene Softwareprogramme zur Verfügung wie beispielsweise das frei verfügbare ImageJ des National Institute of Health (Bethesda, MD, USA) und Sliceomatic der Firma TomoVision, Montreal, Quebec, Canada.

Im Rahmen der Sarkopeniediagnostik wird die Gewebeverteilung lediglich in einer axialen Schnittebene meist auf der Höhe des Wirbelkörpers L3 verwendet. Die Häufigkeit einer computertomographisch diagnostizierten Sarkopenie wird bei Intensivpatient*innen auf 60–70 % geschätzt [73, 74]. Während für spezielle Patient*innengruppen, wie beispielsweise onkologische Patient*innen [75], Patient*innen mit Leberzirrhose [76] oder chirurgische Patient*innen [77], bereits gute Daten vorliegen, steht der breite Einsatz radiologischer Verfahren für die Diagnostik der Körperzusammensetzung bei Intensivpatient*innen jedoch noch am Anfang. Auch bei diesem Verfahren sind jedoch möglicherweise Interferenzen durch einen variablen Hydratationszustand mit zu berücksichtigen [78].

Aus Strahlenschutz- und Kostengründen sollten CT-Untersuchungen nur dann Anwendung finden, wenn sich aus der klinischen Situation heraus eine Indikation für eine solche Untersuchung ergibt und eine entsprechende Expertise zur Auswertung der Körperzusammensetzung lokal verfügbar ist. Die logistischen Argumente gelten ebenso für die MRT. Die Autoren wünschen sich im radiologischen Fachgebiet eine vermehrte Akzeptanz dieser Technik zur Analyse der Körperzusammensetzung; in diesem Zusammenhang ist auch eine automatisierte, beschleunigte Auswertung und Entscheidungsunterstützung durch künstliche Intelligenz wünschenswert [79].

#### 5.5.3. Bioelektrische Impedanzanalyse

Die nicht-invasive bioelektrische Impedanzanalyse (BIA) zielt auf die elektrische Leitfähigkeit des Körpers ab, die durch Messung des Gesamtkörperwiderstands (Impedanz) beim Anlegen eines Wechselstroms (50 kHz bei 0,8 mA) mittels auf Hand- und Fußrücken aufgebrachter Elektroden bestimmt wird. Die Körperimpedanz ist umgekehrt proportional zum elektrolythaltigen Körperwassergehalt (Ohm-Widerstand) und abhängig von den Körperproportionen (d. h. dem Querschnitt des Leiters). Zellmembranen wirken beim Anlegen eines Wechselstroms wie ein Kondensator und verursachen den kapazitativen Widerstand. Hierbei entsteht durch eine Phasenverschiebung der Phasenwinkel, bewirkt durch die kondensatorähnliche Wirkung der Zellmembranen [80]. Der Phasenwinkel kann als Summenparameter für die Gewebequalität betrachtet werden, die abhängig ist vom Hydratationszustand, Ausmaß des „capillary leak“ und von der Körpermagermasse. Somit besteht nur eine bedingte Korrelation mit dem Ernährungszustand.

Unter Einbeziehung des Körpergewichts können über verschiedene Algorithmen die fettfreie Masse, Körperzellmasse und Fettmasse berechnet werden [81, 82]. Diese Berechnungen sind jedoch bei Intensivpatient*innen weit weniger aussagekräftig als der gewichtsunabhängige, jedoch alters- und geschlechtsabhängige Phasenwinkel. Bei extremer Verschiebung des Hydratationszustands muss die Messung der fettfreien Masse und Körperzellmasse als sehr unzuverlässig angesehen werden [83]. Die mittels BIA oder bioelektrischer Impedanz-Vektoranalyse (BIVA) gemessene Veränderung des Phasenwinkels ist insgesamt mit einer erhöhten Sterblichkeit assoziiert [42, 84]. Es ist methodisch limitierend, dass für die am Markt existierenden BIA-Geräte unterschiedliche Gleichungen zur Kalkulation der Körperzusammensetzung basierend auf der gemessenen Resistance (R) und/oder Reactance (Xc) erforderlich sind. Der im Stehen gemessene Phasenwinkel weicht vom Phasenwinkel im Liegen ab; ein Problem, das bei kritisch kranken Patient*innen keine Bedeutung hat. Für die Geräte, die im Liegen messen, sind in wenigen Arbeiten nur geringe Abweichungen des Phasenwinkels beschrieben worden. Das Körpergewicht, das neben der Körpergröße als Grundlage zur Berechnung der Variablen in die Software eingegeben wird, sollte idealerweise aktuell zum Zeitpunkt der BIA-Untersuchung gemessen werden. Zusätzlich sollte bei der Verwendung des Parameters „extrazelluläres Wasser (EZW)“ auch eine aktuelle Erniedrigung des Albuminspiegels berücksichtigt werden.

**Figure 1:**
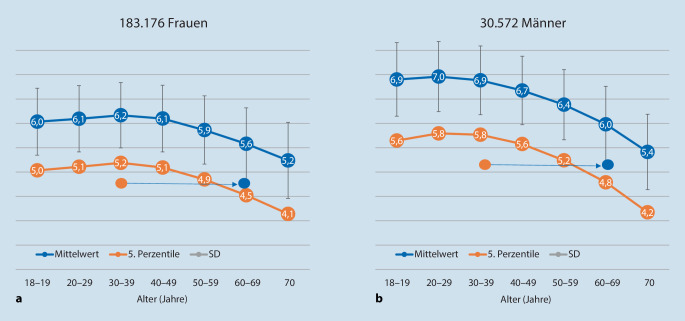

Die Berechnung der Körperkompartimente hat jedoch für die Bestimmung des Ernährungszustands keine Bedeutung. In Bezug auf die Prognose wurde der Phasenwinkel in mehr als 170 Studien an über 40.000 Patient*innen untersucht und hat sich bei den verschiedensten Grunderkrankungen – auch bei Intensivpatient*innen [85, 86] – als aussagekräftig insbesondere im intraindividuellen Verlauf erwiesen. So waren Veränderungen des mittels BIA bestimmten Phasenwinkels bei kardiochirurgischen Patient*innen mit einem verlängerten Aufenthalt auf der ITS und im Krankenhaus assoziiert [87]. Ein niedriger Phasenwinkel wird als prognostisch kritisch beurteilt und kann mit einer erhöhten Sterblichkeit einhergehen [88]. Normwerte und Standardabweichungen für einzelne Alters- und Geschlechtsgruppen liegen vor (Abb. 1). So wird zur besseren Vergleichbarkeit idealerweise der standardisierte Phasenwinkel eingesetzt. Zur Berechnung des standardisierten Phasenwinkels wird der gemessene Wert vom dem für das jeweilige Alter und Geschlecht bekannten Mittelwert subtrahiert und die Differenz durch die entsprechende Standardabweichung dividiert.

Aufgrund der inhaltlichen Besonderheiten des Phasenwinkels ist der Bezug zu Screeningtools für die Diagnose einer Mangelernährung noch unklar. Eine Querschnittsstudie an 55 kritisch kranken COVID-19-Patient*innen ergab, dass zwar ein niedrigerer Phasenwinkel mit einem höheren Risiko verbunden war, eine schwerere COVID-19-Infektion zu entwickeln. Ein direkter Bezug zum Ernährungszustand der Patient*innen konnte jedoch nicht hergestellt werden [89]. Zusammenfassend reflektiert der Phasenwinkel die Qualität der Magermasse nur zum Teil und ist sowohl ein Indikator für eine kataboliebedingte Störung der zellulären Integrität als auch für eine inflammatorisch bedingte Hyperhydratation („capillary leak“). Die prognostischen Fähigkeiten des Phasenwinkels sind jedoch denen der anderen BIA-Parameter überlegen [90].

##### Empfehlung 4

Empfohlen wird bei Intensiv- und Intermediate Care-Patient*innen eine nicht-invasive serielle Untersuchung der Skelettmuskelmasse zur Erkennung und Verlaufskontrolle kataboler Veränderungen des Ernährungszustands. Geeignete Verfahren sind Sonographie/BIA//CT. Interferenzen durch einen variablen Hydratationszustand müssen dabei berücksichtigt werden. Bei Hochrisikopatient*innen mit zu erwartender längerer Verweildauer (z. B. NUTRIC-Score > 5) kann eine Bestimmung des Phasenwinkels mittels BIA hilfreich sein.

## 6. Zusammenfassung

Offen ist die Frage, ob bei Risikopatient*innen mit einer personalisierten und optimierten MNT, die an den Ernährungsstatus bei Aufnahme und im Verlauf angepasst ist, ein Muskelabbau komplett verhindert (und nicht nur minimiert) werden kann, um so das klinische Outcome zu verbessern. Unklar ist bis heute ebenfalls die Optimierung der MNT im Hinblick auf immunologische und reparative Funktionen [4]. Grundvoraussetzung einer effektiven MNT ist jedoch immer, durch kausale Therapie die Signalketten zu unterbrechen, die die Katabolie von Seiten des inflammatorischen/infektiösen Fokus her auslösen und unterhalten [91].

Im Hinblick auf die apparative Ausstattung der ITS oder IMC sollten entsprechend der DIVI-Strukturempfehlungen zur Erfassung des Ernährungsstatus eine Bett- bzw. bei mobilisierbaren Patient*innen eine Personen‑/Stuhlwaage (1C) und ein Sonographiegerät (2C) obligat verfügbar sein [5, 6]. Die Expertengruppe empfiehlt aufgrund der günstigen Anschaffungskosten (ca. 200 €) zusätzlich ein Dynamometer (Tab. 3). Eine bioelektrische Impedanzanalyse ist fakultativ. Eine spezifische apparatetechnische Diagnostik sollte speziell bei Hochrisikopatient*innen (vgl. oben) nach der klinischen Stabilisierung regelmäßig (einmal wöchentlich) erfolgen.

**Table 3:** 

Obligat	Fakultativ
Bettwaage^a^ ggf. mit Hebevorrichtung bzw. Personen‑/Stuhlwaage bei möglicher Mobilisation	Bioelektrische Impedanzanalyse
Sonographiegerät ^b^	
Dynamometer	

DIVI-Strukturempfehlungen: ^a^1C, ^b^2C [5, 6]

Ferner verweisen wir darauf, dass für die im Anhang zusammengestellten Anforderungen für die Kodierung der ernährungsmedizinischen Komplexbehandlung „OPS 8-98j“ gemäß aktuellem aG-DRG Fallpauschalenkatalog die Erhebung des Ernährungsstatus bei Aufnahme und die apparative Verlaufskontrolle mit Handkraftmessung und bioelektrischer Impedanzanalyse oder indirekter Kalorimetrie einmal wöchentlich unabdingbare Voraussetzung sind.

Auf die Messung des Ruheenergieumsatzes sowie auf laborchemische Parameter zur Beurteilung des Ernährungsstatus und der metabolischen Toleranz wird in einem weiteren Positionspapier eingegangen.

### 6.1 Vorgehen in der klinischen Praxis

**Figure 2:**
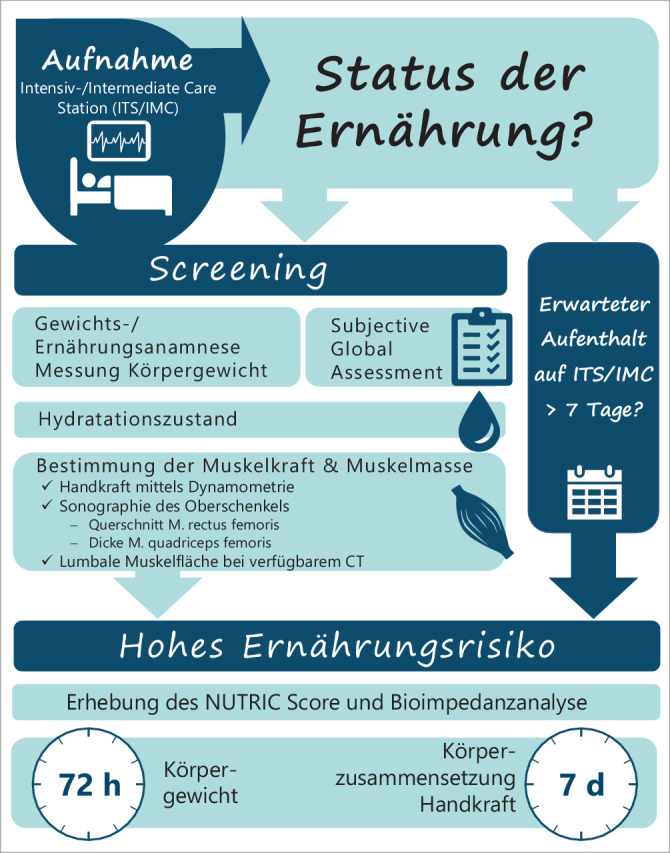

Die Erfassung des Ernährungsstatus erfolgt in mehreren Schritten (Abb. 2):

1. Bei Aufnahme auf die ITS oder IMC sollten als Basis bei wachen, ansprechbaren und auskunftsfähigen Patient*innen eine genaue Gewichts- und Ernährungsanamnese erhoben werden. Empfohlen wird die Durchführung eines SGA sowie einer Handkraftmessung. Bei nicht anamnestizierbaren Patient*innen sollte wenn möglich eine Fremdanamnese erfolgen. Ist das aktuelle Körpergewicht nicht bekannt bzw. nicht eruierbar, sollte dieses mittels Bettwaage (oder Personen-/Stuhlwaage bei möglicher Mobilisation) gemessen werden. Zusätzlich sollte der Hydratationszustand mittels körperlicher und/oder sonographischer Untersuchung festgestellt werden. Es sollte geklärt werden, ob eine zeitnahe CT-Untersuchung des Abdomens vorliegt; falls ja, sollte nach Rücksprache mit geschulten radiologischen Kolleg*innen die lumbale Muskelfläche auf Höhe LWK 3 bestimmt werden. Sind die technischen Voraussetzungen gegeben, sollte zusätzlich möglichst innerhalb von 48 h eine Sonographie zur Bestimmung der Dicke des M. quadriceps und/oder Querschnitts des M. rectus femoris erfolgen.
2. Bei einem voraussichtlichen Aufenthalt > 7 Tagen auf der ITS oder IMC, bei einem NUTRIC-Score > 5 oder einem Phasenwinkel < 4,0 sollten serielle Untersuchungen erfolgen. Bei diesen Patient*innen sollte mindestens zweimal wöchentlich eine Messung des Körpergewichts sowie einmal pro Woche eine Verlaufskontrolle der Körperzusammensetzung mit einem der angegebenen Verfahren (BIA, Sonographie, CT nur bei klinischer Indikation) und der Handkraft mittels Dynamometrie erfolgen; dabei ist Voraussetzung, dass Veränderungen des Hydratationszustands in die Interpretation der Befunde mit einbezogen werden bzw. entsprechende Korrekturen erfolgen.

### Supplementary Information





## Anhang

### Ernährungsmedizinische Komplexbehandlung 8-98j

https://www.dimdi.de/static/de/klassifikationen/ops/kode-suche/opshtml2022/block-8-97...8-98.htm

Hinweis: Diese Codes sind auch bei intensivmedizinisch versorgten Patient*innen anzugeben. Die Art der Ernährungstherapie als medizinische Hauptbehandlung ist gesondert zu codieren (8-015 ff., 8‑016). Die Art der Ernährungstherapie als medizinische Nebenbehandlung ist bei nicht intensivmedizinisch behandelten Patient*innen gesondert zu codieren (8-017 ff., 8‑018 ff.)

- Strukturmerkmale:
  - Ernährungsteam mit Behandlungsleitung durch einen Facharzt mit der strukturierten curricularen Fortbildung oder Zusatzbezeichnung Ernährungsmedizin und mit einem Diätassistenten oder Ökotrophologen
  - Werktags mindestens 7‑stündige Verfügbarkeit des Ernährungsteams
- Mindestmerkmale:
  - Standardisiertes Screening des Ernährungsstatus innerhalb der ersten 48 h nach stationärer Aufnahme (z. B. NRS 2002, MNA oder NUTRIC Score)
  - Standardisiertes ernährungsmedizinisches Basisassessment zu Beginn der Behandlung durch ein Mitglied des Ernährungsteams, bestehend aus:
    a) Ernährungsanamnese inkl. aktueller Nahrungsaufnahme
    b) Handkraftmessung
    c) Bestimmung der Körperzusammensetzung mittels Bioimpedanzanalyse oder Bestimmung des Energieumsatzes mittels indirekter Kalorimetrie
    d) Energie- und Nährstoffbedarfsermittlung unter Berücksichtigung von Verträglichkeit und Gesamtbilanz
  - Erstellung eines individuellen Behandlungsplans (oral, Trinknahrung, enteral und/oder parenteral nach einem Stufenschema der Ernährung) zu Beginn der Behandlung
  - Mindestens 2‑mal pro vollständiger Woche Verlaufs- und Zielkontrolle der dokumentierten Nahrungsaufnahme (oral, Trinknahrung, enteral und/oder parenteral), davon einmal mit Durchführung folgender Verfahren:
    a) Handkraftmessung oder Bioimpedanzanalyse oder indirekte Kalorimetrie
    b) Erfassung von Gewicht/Body-Mass-Index
  - Wöchentliche Teambesprechung
  - Untersuchungen, wie z. B. Body-Mass-Index oder Handkraftmessungen, sind entbehrlich, wenn sie aus medizinischen Gründen (Amputationen, Lähmungen, Sedierung o. Ä.) nicht durchführbar sind
  - Indikationsabhängige Empfehlungen für den weiterversorgenden Arzt und/oder Homecare-Dienstleister
- 8‑98j.0 Bis zu 6 Behandlungstage
- 8‑98j.1 Mindestens 7 bis höchstens 13 Behandlungstage
- 8‑98j.2 Mindestens 14 bis höchstens 20 Behandlungstage
- 8‑98j.3 Mindestens 21 Behandlungstage
